# Neural correlates of visualizations of concrete and abstract words in preschool children: a developmental embodied approach

**DOI:** 10.3389/fpsyg.2015.00856

**Published:** 2015-06-29

**Authors:** Amedeo D’Angiulli, Gordon Griffiths, Fernando Marmolejo-Ramos

**Affiliations:** ^1^Department of Neuroscience, Carleton University, Ottawa, ONCanada; ^2^Institute of Interdisciplinary Studies, Carleton University, Ottawa, ONCanada; ^3^Neuroscience of Imagery Cognition and Emotion Research Lab, Carleton University, Ottawa, ONCanada; ^4^Gösta Ekman Laboratory, Department of Psychology, Stockholm University, StockholmSweden

**Keywords:** embodied cognition, preschool children, word processing, ERPs, visual mental imagery, visualization

## Abstract

The neural correlates of visualization underlying word comprehension were examined in preschool children. On each trial, a concrete or abstract word was delivered binaurally (part 1: *post-auditory visualization*), followed by a four-picture array (a target plus three distractors; part 2: *matching visualization*). Children were to select the picture matching the word they heard in part 1. Event-related potentials (ERPs) locked to each stimulus presentation and task interval were averaged over sets of trials of increasing word abstractness. ERP time-course during both parts of the task showed that *early* activity (i.e., <300 ms) was predominant in response to concrete words, while activity in response to abstract words became evident only at *intermediate* (i.e., 300–699 ms) and *late* (i.e., 700–1000 ms) ERP intervals. Specifically, ERP topography showed that while early activity during post-auditory visualization was linked to left temporo-parietal areas for concrete words, early activity during matching visualization occurred mostly in occipito-parietal areas for concrete words, but more anteriorly in centro-parietal areas for abstract words. In intermediate ERPs, post-auditory visualization coincided with parieto-occipital and parieto-frontal activity in response to both concrete and abstract words, while in matching visualization a parieto-central activity was common to both types of words. In the late ERPs for both types of words, the post-auditory visualization involved right-hemispheric activity following a “post-anterior” pathway sequence: occipital, parietal, and temporal areas; conversely, matching visualization involved left-hemispheric activity following an “ant-posterior” pathway sequence: frontal, temporal, parietal, and occipital areas. These results suggest that, similarly, for concrete and abstract words, meaning in young children depends on variably complex visualization processes integrating visuo-auditory experiences and supramodal embodying representations.

## Introduction

### Visualization and Abstract/Concrete Word Acquisition

Understanding the way we mentally simulate the meaning of concrete and abstract words constitutes a critical test for the evaluation of theories according to which language is grounded in embodied cognition. These theories involve the interplay of perception, action, and socio-emotional systems (Scorolli et al., 2011). Visual mental imagery, defined as the brain and mental processes like those that arise during visual perception but in the absence of appropriate immediate visual stimuli in the retina (D’Angiulli and Reeves, 2007), represents a major related and parallel challenge in addressing the question of how mental representations become part of cognitive activity for action and language (Paivio, 2007). Although originated from a different stream of research within experimental cognitive psychology, the theoretical construct of visual mental imagery can be reformulated in terms of the newer embodied cognitive research field. That is, visual simulation (also called visualization) consists of a re-enactment of the perceptual, motor, and introspective states acquired during sensory and perceptual experience with the world, body, and mind (Barsalou, 1999). Although there is agreement that the abilities underlying visualization play an important role for children’s social and cognitive development (Sadoski and Paivio, 2007) and, more recently, an interest in understanding how vision plays a key role in children’s embodied cognition (e.g., D’Angiulli and Schibli, 2011; Iossifova and Marmolejo-Ramos, 2013; also see D’Angiulli, 2011), much research is needed to advance research results and theories. The purpose of the present work was to explore the neural correlates of visualization of concrete and abstract words in preschool children. In so doing, we attempted to merge the classic neurophysiological approach to imagery with visualization as studied in the embodied cognition field.

According to Paivio’s (2007) classic and influential dual coding theory (DCT), concrete words are represented in two systems, imagery and linguistic, whereas abstract words are represented solely in the linguistic system. In contrast, for some embodiment models, both concrete and abstract words are grounded in perception and action systems, even if the linguistic system plays a major role for abstract words. Thus, for example, some embodied models (Borghi and Cimatti, 2009; Dove, 2011, 2014; Borghi and Binkofski, 2014) argue that when comparing the words “bicycle” and “justice,” “bicycle” would activate more perceptual and motor experiences, whereas “justice” would activate less perceptual and motor experiences but more linguistic experiences than “bicycle” (Glenberg et al., 2004). This type of embodiment, known as graded embodiment, assumes that low or high activation of sensorimotor systems can occur during language processing and that the task and the stimuli *per se* play a key role in such activation (e.g., Chatterjee, 2010; Meteyard et al., 2012; Marmolejo-Ramos and Dunn, 2013).

The research questions concerning concrete and abstract word comprehension may best be framed using models that emphasize vision-language interaction. Synthesis among different processing modality channels may occur through mapping between linguistic and object representations in the visual world. Various candidate mechanisms, with evidence empirically supporting representational mapping, include anticipatory eye movements, memory, and attention processes occurring during comprehension and visual sentence matching tasks (see Huettig et al., 2011 for a review). These mechanisms could reflect the embodiment of language and vision synthesis, not “representations” postulated in DCT or similar models. Although cognitive in nature, these embodied mechanisms may provide the substrate for words as “social tools” (Borghi and Cimatti, 2009; see also Granito et al., 2015). Most recently, it has been argued that the embodied cognition framework provides a platform for the social foundation of word acquisition. In particular, Borghi et al. (2013) propose that abstract word meanings depend on exposure in everyday experience to language in social contexts to a greater extent than concrete word meanings. From this view, the difference between concrete and abstract words depends on the different modes of acquisition, which can be perceptual, linguistic, or mixed, or can change with age, schooling, and more in general with social interaction with adults and peers (see Schwanenflugel, 1991; Wauters et al., 2003).

As noted by Mishra and Marmolejo-Ramos (2010), a current limitation is that most vision-language interaction accounts generally do not explicitly address the role of embodied simulations and the interplay between sensory-perceptual and memory systems. Improving on current proposals, those authors presented the idea of a highly dynamic interactive model, here called the dynamic interaction vision-language approach (DIVLA), wherein “mental representations built during vision-language interaction affect both perception and action at both a behavioral (events) and neurological (systems) level” (Mishra and Marmolejo-Ramos, 2010, p. 301). Different from other proposals, DIVLA proposes that visual components have primacy over motor components in terms of supporting the enacted or re-enacted (memory) simulations that mediate mapping between language and the visual world. It is worth noting that other sensory modalities can be associated with language processing (e.g., the auditory system and its role in phonological processing); however, the brain structures devoted to visual processing and the detrimental effects on overall cognitive processing when visual areas are damaged indicate that vision is a privileged system in human cognition (Givón, 2002).

The DIVLA model contends that (i) visual inputs influence motor systems, (ii) linguistic inputs affect motor events, and (iii) situation models generated during the vision-language interaction have an effect on sensorimotor systems (see Mishra and Marmolejo-Ramos, 2010, p. 301). That is, the model is similar to current embodied cognition theories (e.g., Wilson, 2002; Feldman and Narayanan, 2004; Meteyard et al., 2012); but with a larger emphasis on the vision-language interface. Additionally, and although not explicitly stated in the original proposal, the model also assumes graded embodiment such that the level of activation of sensory and/or motor systems is task and stimuli dependent (e.g., Marmolejo-Ramos and Dunn, 2013; Arbib et al., 2014). Such an approach is in line with metaphorical mapping proposals (e.g., Lakoff, 2014) in that the comprehension of abstract concepts relies on their association to concepts that are less abstract and that rather favor concreteness. Therefore, some concepts might have very low levels of sensorimotor activation; whereas other concepts can show high levels of embodiment (e.g., Xue et al., 2015; see Siakaluk et al., 2008, for evidence of categorizing concrete words according to the level of body-object interactions). The ultimate goal is the production of mental representations that include proxies of the perceptual and motor events associated with the concept being learned or evoked. Current advances in social cognition bring to the forefront the idea that social interactions play a key role in forming such mental representations (see Marmolejo-Ramos and D’Angiulli, 2014).

The principles of DIVLA have also been extended to account for social factors influencing embodied cognition. Specifically, the new account argues that shared embodied situation models are constructed during spontaneous discourse by adding kinesics (e.g., gestures) and paralinguistic (e.g., prosody) factors to language comprehension and production (Cevasco and Marmolejo-Ramos, 2013). That is, various perceptual components associated with concepts (e.g., sight of the actual object) are presented along with prosodic (e.g., pronunciation of the object’s name) and body (e.g., hand movements that further describe the object) components. The embodied situation model is supported by developmental embodied theories arguing that physical, social and linguistic inputs are vital in the acquisition of knowledge based on sensorimotor interactions with the environment (see Smith and Gasser, 2005).

For over 30 years, Paivio et al. (1968) and Clark and Paivio (2004) have validated, for a large corpus of English words, the link between the continuum of concreteness/abstractness and ease/difficulty for imagery or image-ability (i.e., the self-rated ease with which a participant can imagine the object or concept corresponding to a word). Such prior research forms the foundation for studying visualization of words in preschool children because, currently, there is no unifiable, validated, and reliable word database for the preschool population. One first step toward acquiring a preschool database is the assessment of electrophysiological and behavioral linguistic categorization. Preschool linguistic categories are acquired by listening to other people explain a word’s content which involves differing degrees of complexity during word acquisition. Therefore, the use of linguistic labels may be crucial for merging experiences in social contexts. With respect to the earlier example, learning to use the word “bicycle” is easier than learning to use the word “justice” because the notion of “justice” must be acquired socially. Note that visualization can be triggered by linguistic input that can come in the form of visually or aurally presented words. For example, it has been shown that there are separate input paths for words presented visually (reading) vs. words presented aurally (listening) (Gleason and Bernstein, 1998).

Previous research with adults has typically shown concrete words elicit a greater N400 than abstract words at posterior sites (Holcomb et al., 1999; West and Holcomb, 2000; Lee and Federmeier, 2008; Tsai et al., 2009) as well as a later negativity for concrete vs. abstract words (Holcomb et al., 1999; West and Holcomb, 2000; Lee and Federmeier, 2008; Huang et al., 2010); however, these tasks often use sentence presentation with the last word presented as either concrete or abstract. Welcome et al. (2011) performed a single word processing study in which participants were required to either generate word associations or mental images. Participants elicited the same increased negativity (N400) as previous studies for concrete words during word association but, contrary to previous studies, during the imagery condition, abstract words elicited a more negative response at 756–1500 ms. To better localize the effects of word concreteness and task demands, Gullick et al. (2013) modified West and Holcomb (2000)’s procedure by presenting single words in two conditions, one requiring visualization and imagery and the other requiring simple letter search. Gullick et al. (2013) found, similar to previous research, that the N400 was sensitive to concreteness and visualizability and that it extended from temporo-parietal to frontal medial sites. However, the concreteness effect was also observed for the simple letter search possibly suggesting that concreteness may not depend on word meaning during this type of single word processing task. The concreteness effect associated with simple letter search, it was argued, may be due to the lack of sentential context. The visualization concreteness effects, on the other hand, were task dependent and, for the N400, were found to have the greatest amplitudes at medial temporo-parietal sites. Therefore, those authors suggested that concreteness and visualization are discrete processes quantified by the N400. Their findings for the later N700 indicated primarily frontal activity which was attributed to mental imagery processing for concrete words.

The studies that we just reviewed, similar to fMRI experiments, approach the processing of concrete and abstract words as a “tip of the iceberg” problem; that is, as static events captured at specific moments in the ERP (i.e., N400) at specific electrode sites. Accordingly, the methods employed aim to determine which electrodes to select and statistically analyze as single separable outcomes, and by design, they only partially address the underlying neurocognitive dynamical processes. The focus of interest in the present study was to examine how sensory visualization supports young children’s acquisition of word meaning and implicates a network of connections overlapping dynamically within the visuospatial and language pathways. Using children data, we employed a novel approach to examine concrete and abstract word/picture processing as a post-stimulus onset neural path progression from the onset pairing of the auditory word and the visuo-auditory word-picture matching up to the moment of the selection of the correct picture.

### Study Design and Hypotheses

**FIGURE 1:**
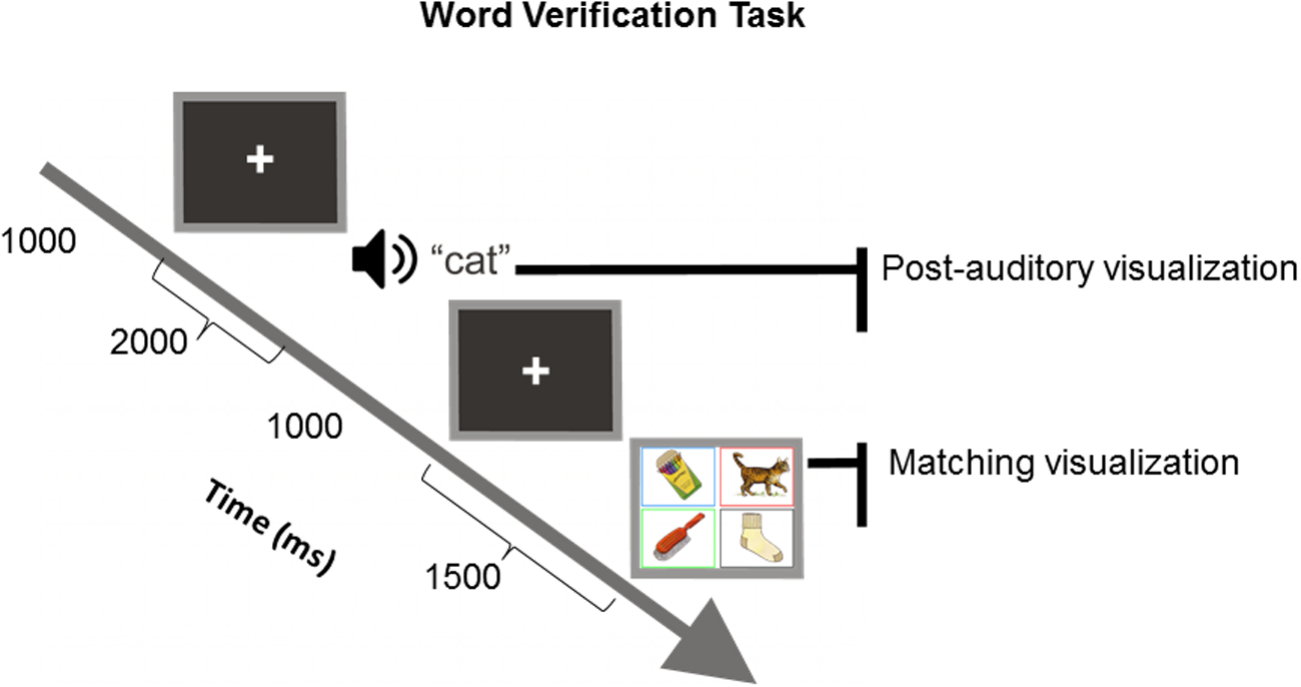
**Representation of the word verification task (WVT) used in the present experiment, indicating stimulus timing for the auditory and visual parts of the task.** The interval for the auditory and visual averages was -200 to 1000 ms around stimulus onset followed by a correct response to the visual stimulus. The visual presentation remained on the screen until the participant made a response. The length of the post-response cross fix allowed the researcher to re-iterate the experiment requirements to the participant (e.g., “try not to blink, remain still,” etc.) if necessary.

Auditory and visual stimuli were used in a two-part word verification task (WVT, see **Figure 1**) to examine the neural correlates of visualization underlying word comprehension in preschool children. It is reasonable to assume that because preschool children do not have a fully developed sophisticated linguistic system yet, they are likely to experience a certain degree of difficulty understanding intricate concepts involving time, weight, or size or the use of prefixes and suffixes (e.g., Jones and Smith, 1993), therefore, only nouns and verbs were used in WVTs. In the following experiment, the processing underlying the first part of our task corresponded to automatic sensorimotor system activation (Gallese, 2009) during the aural presentation of single words (WVT-A) and was assumed to represent the initial imagery component associated with listening and comprehending words, which we nickname “post-auditory visualization.” The second part corresponded to activation of larger systems (Decety and Grèzes, 2006) including sensorimotor areas in the dorsal and ventral visual pathways during visual presentation (WVT-V) of a picture array and was assumed to represent the attention, re-enactment of previous experience of the word heard and word-picture match decision component (with pictures corresponding to the previous aurally presented word); this latter complex process was nicknamed “matching visualization.”

Event-related potentials (ERPs) measured the neuro-electrophysiological activity concomitant with auditory word and image verification parts and can capture the differences between the two parts. ERPs are electroencephalographic (EEG) waveforms averaged over specific intervals and are time-locked to the presentation of a stimulus or other task component. The recorded electrical brain activity changes in reference to a pre-stimulus baseline with millisecond (ms) temporal resolution. Particular relevance was given to two well-established temporal intervals. The first “early” interval occurs from 0 to 299 ms and reliably reflects changes in brain activity related to visual and auditory perceptual processing (Hillyard and Picton, 1987; Freeman, 1991; Luck, 2005; Schomer and da Silva, 2010). The second “late” interval occurs from 700 to 1000 ms, and reflects changes in brain activity related to semantic processing (Sitnikova et al., 2008) and imagery (Freeman, 1983; Farah et al., 1989; Andreassi, 2007; Sitnikova et al., 2008). Examination of the intermediate interval from 300 to 699 ms encompasses the P300 decision-making processes and the N400 concreteness effect found in previous research we reviewed earlier.

Recordings were made from surface electrodes positioned in accordance with the universal standard of electrode placement (Klem et al., 1999). Two complementary systems of neurocognitive network models were adopted as guiding categories in order to relate task performance and ERP activity to underlying functions and systems in the developing brain of preschool children.

The first simplified adaptation of brain subdivision (from Coch et al., 2005; Hackman and Farah, 2009) and electrode site correspondence (from Stevens et al., 2008) were considered as components of a functional system organized into four relatively distinct neurocognitive networks representing *domain-general cognitions* (Gelman, 2001). The proposed neurocognitive networks were derived from clinical neuropsychological or neuroanatomical studies based on functional neuroimaging studies in healthy children while they performed similar or related cognitive tasks (for an extended rationale see Noble et al., 2005). The networks included: (1) Pre-Frontal and Frontal Executive corresponding to F electrode sites F3, Fz, F4; (2) the Centro-Temporal-Memory corresponding to C-T electrode sites T7, Cz, T8; (3) the Parietal-Spatial-Cognitive corresponding to P electrode sites P7, Pz, P8 and (4) the Occipital-Visual-Cognitive corresponding to O electrode sites O1, Oz, O2.

**FIGURE 2:**
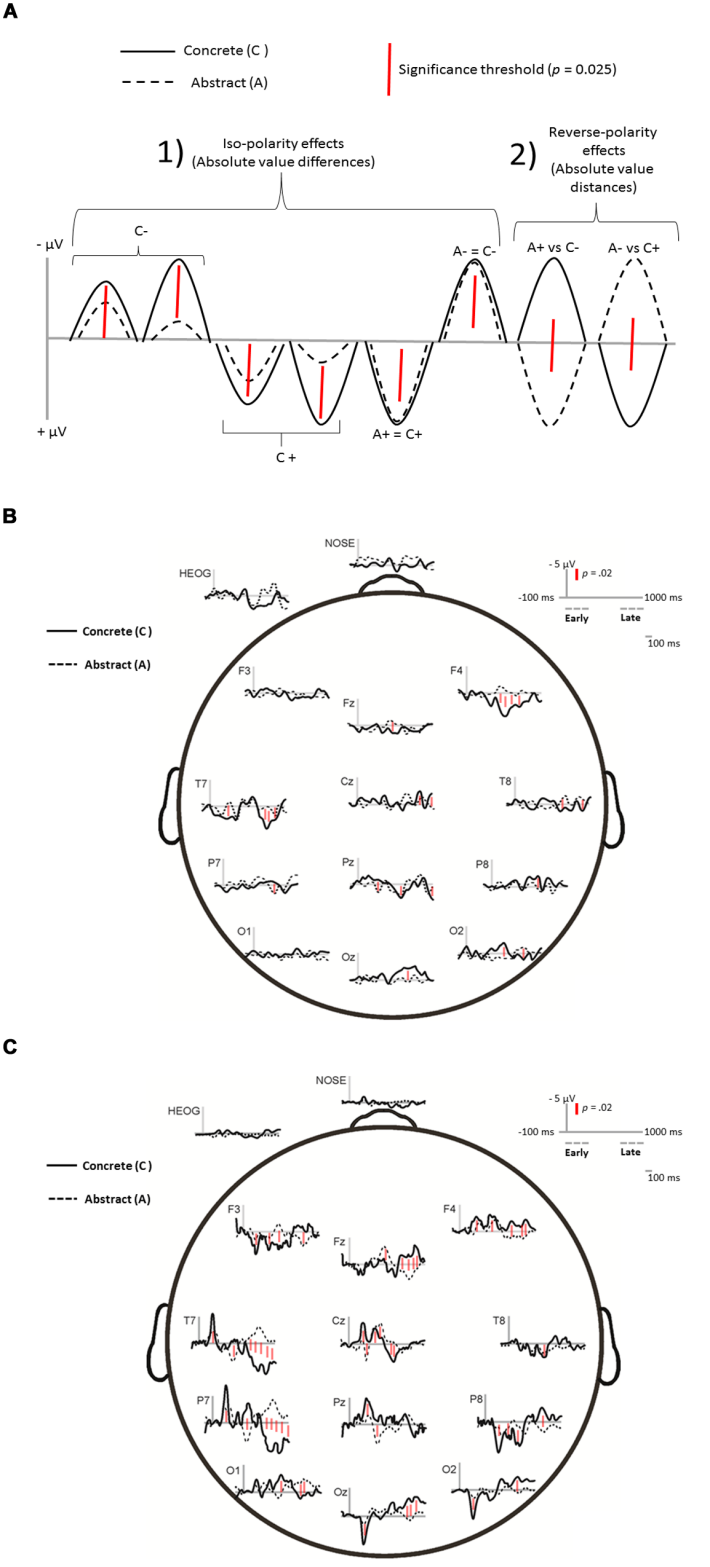
**(A)** Study of waveform differences and homologies empirically observed for ERP activity in response to concrete and abstract words. To quantify distances shown in the waves, the Iso-polarity effects involved computing the differences in absolute values, whereas reverse polarity effects involved computing the sum of absolute values. The significance threshold shown as red distance-bar was corrected for multiple comparison testing using the Simes–Bonferroni procedure (see text). Grand average ERP waveforms for concrete and abstract words conditions in the auditory **(B)** and visual parts **(C)** of the WVT. The magnitude of the minimum significant effect is represented as a red distance-bar (drawn in scale with respect to 5 μV). Distances/differences larger than the red bar were significant at least at *p* < 0.05, as examples, main points of significance are marked with the red bar in the waveforms.

The same electrodes used in the above-mentioned were simultaneously considered as components of a system within another functional organization, that represented *domain-specific cognitions* (Gelman, 2001), including three broader, higher-level, networks that were represented by a subgrouping of electrodes functionally contingent on the lateralization shown in the recorded activity. Therefore, activity in any of the four networks described for the domain general system could at the same time be referred to more broadly as (1) the *Left* Perisylvian/Linguistic Working Memory network, corresponding to odd numbered electrode sites, O1, P7, T7, F3, (2) the *Right* Perisylvian/Spatial Working Memory network corresponding to even numbered electrode sites, O2, P8, T8, F4 (for extensive rationale see, Gazzaniga, 2009), and (3) the *Midline* or Attentional network (Posner et al., 2007) corresponding to mid-electrode sites, Oz, Pz, Cz, Fz from posterior to anterior regions (see **Figure 2**).

In the context of the proposed framework, since activity recorded in each electrode could represent at the same time the engagement of both systems (given that electrodes record the activity of millions of neurons and confound different networks at the same time), the ensuing analysis had to be logically framed in terms of possible ‘Domain general × Domain specific’ interactions. That is, in order to interpret the real meaning of the activity of each electrode, it was not sufficient to examine electrodes as groups corresponding just to either domain general or domain specific processes. We needed to identify the specific waveforms occurring in each electrode in order to have a finer grained perspective of the electrodes’ activity, possibly expressing such important functional interaction. Accordingly, the present approach is logically linked with the following hypotheses and subsequent data analysis.

Consistent with graded embodiment and social cognition theories, it is reasonable to hypothesize that if abstract words do not have a specific object or entity referent, they may be acquired socially through linguistic rather than perceptual labeling. Linguistic labeling is acquired by listening to other people explain the word content and involves different degrees of complexity during word acquisition. As for the earlier example, learning to use the word “bicycle” is easier than learning to use the word “justice.” The use of linguistic labels may be crucial for merging experiences in social communication contexts in order to interpret the notion of “justice.” It is important to note that difficulty or effort in word processing should not be regarded as confound, but rather it reflects the traditional operationalization of the construct of ‘abstractness’ in experimental psychology for behavioral measurement of semantic processing and it covaries with visualization (see Paivio, 2007).

Based on graded embodiment theories, we predicted that abstract (i.e., relatively more difficult, more effortful) and concrete (i.e., relatively easier, less effortful) words should both evoke visualizations grounded in perception and action. For the matching visualization (i.e., the imagery component of the WVT associated with the visual stimuli, pictures corresponding to words) we expected to observe early ERPs similar to those observed for the post-auditory visualization (i.e., the imagery component of the WVT associated with listening and comprehending words presented aurally), showing similar posterior occipito-parietal (O-P) activity for both the concrete and abstract words, mainly along the midline attentional networks.

However, according to the DIVLA hypothesis – and differently than in most other theories – during the post-auditory visualization, we expected early and late ERPs to show different patterns of interactive visual-spatial-language network activation, for both the concrete and abstract words. Specifically, early ERPs may show relatively more abstractness effects in the right occipital-parietal networks (O-P), whereas left Frontal (F) networks may show relatively more ‘concreteness’ effects. Conversely, late ERPs may show a pattern inverse to that shown for the early ERPs, with more ‘abstractness’ in the right O-P and more ‘concreteness’ in left F.

## Materials and Methods

### Participants

Participants were selected from a cohort of children recruited in the context of a separate, larger, non-overlapping research program on early development screening (D’Angiulli et al., 2009). An information package was distributed to all parents whose children attended the same daycare of a middle-sized city in British Columbia to recruit preschool children. Small information sessions were provided periodically at the daycare to keep parents informed. Only general information regarding the present study was provided to target families and children. All parents provided signed informed consent before allowing the child to participate in the study, and then completed questionnaires on familial demographic and socioeconomic information. Prospective participants were contacted following screening of the information provided in the questionnaires and daycare records. Of the 30 families contacted by mail, 17 returned completed signed consent for the present study. Upon arrival at the research center, child’s active assent was obtained according to protocols approved by the Research Ethics Boards of all participating institutions. Children were given a $5 gift card for participation and a book of stickers at the end of their session. The study hypotheses and purposes were provided verbally to the child and in writing to the parents during debriefing but not during recruitment to prevent any participant bias.

From the original sample of 16 (one child who initially signed up withdrew from the study), a final sample of 13 children [Male = 9; *M* age (SD) = 5.12 (0.75); right-handed = 11] was obtained after excluding three (female) participants due to insufficient artifact-free usable EEG data (*n* = 2) and performance values outside the norm (*n* = 1). As a result of applying strict inclusion criteria, selected participants represented a relatively homogeneous group of healthy, typically developing children. All were Caucasian with normal or corrected-to-normal vision and no hearing or other known sensory impairments. The children lived in the same neighborhood corresponding to the same catchment area for the daycare center they attended. All children were from middle-high family socioeconomic background as defined by Statistics Canada norms (Statistics Canada, 2001). The particular recruitment setting was selected for its known, well-documented socio-geographic characteristics (Kershaw et al., 2005).

Parents reported English as the child’s first or second language with fluency in English if English was not the first language. Children’s scores for the *Behavioral Rating Inventory of Executive Function – Preschool Version* (Gioia et al., 2009), *Child Behavior Checklist for Ages 1.5–5 (CBCL/1.5–5;* Achenbach, 2009), *Caregiver-Teacher Report Form for Ages 1½–5* (Achenbach, 2009), Executive Function assessment across seven traits showed all participants within normal limits (see **Table 1**). Furthermore, the participants were typically developing children with no history of medication or referral to disability assessment or services as ascertained from parent report and daycare records.

**Table 1:** Sample characteristics showing mean and median scores for general demographics and screening questionnaires.

Descriptive statistics							

**Variables**	**Female**	**Male**	**Mean**	**Median**	**Median range**	**SD**	**SE**
Gender	4.00	9.00					
Age			5.12	5.00		0.75	0.87
Income (in 1000s)				70507.90			
Executive function^a^				38.50	26.45–71.36	27.16	1.29
CBCL^b^							
Emotionally reactive			56.50			15.26	5.40
Anxious/depressed			56.75			14.38	5.08
Somatic complaints			58.38			15.00	5.31
Withdrawn			60.38			12.94	4.58
Sleep problems			61.63			21.54	7.62
Attention problems			53.75			7.59	2.68
Aggressive behavior			54.63			7.58	2.68
Internal computations tscores			46.63			8.52	3.01
External computations tscores			44.87			7.12	2.52
Total problems tscores			43.38			10.54	3.73
Affective problems			60.25			18.78	6.64
Anxiety problems			50.50			1.41	0.50
Pervasive developmental problems	55.75			10.44	3.69		
Attention deficit/hyperactivity problems	51.00			2.83	1.00		
Oppositional defiant problems		60.63			12.82	4.53	
TRF^c^							
Emotionally reactive			50.56			21.61	7.20
Anxious/depressed			58.89			10.54	3.51
Somatic complaints			52.78			8.33	2.78
Withdrawn			56.33			10.98	3.66
Attention problems			55.56			13.78	4.59
Aggressive behavior			60.22			16.39	5.46
Internal computation tscores			46.44			7.52	2.51
External computation tscores			44.56			10.88	3.63
Total problems tscores			44.56			9.99	3.33
Affective problems			56.11			15.19	5.06
Anxiety problems			59.44			11.83	3.94
Pervasive developmental problems	58.11			11.56	3.85		
Attention deficit/hyperactivity problems	57.22			13.93	4.64		
Oppositional defiant problems		60.22			16.56	5.52	
Denver developmental screening test^d^		65.90			36.71	11.61	

*^a^Executive Function assessment was based on scores from Emotional Control, Working Memory, Planning and Organization, Self-Control Index, Flexibility Index, Emergent Metacognition Index, and Global Executive Composition, ^b^Child Behavior Check List percentile score, ^c^Caregiver-Teacher Report Form percentile score, ^d^Denver Developmental Screening Test or Denver Scale scores. All Denver subscales (i.e., Personal – Social, Fine Motor – Adaptive, Language, Test Behavior – Attention Span, Articulation and Intelligibility) were within 1 SD from the mean.*

### Materials and Procedures

#### Word Verification Task

The WVT is a computerized version of the PPVT-III^1^ which includes a total of 19 word sets with 24 target words (see Supplementary Material Table S1) per set presented aurally followed by a corresponding colored four-picture array (see **Figure 1**). Eight practice trials were initially presented and participants were asked to identify two concrete words by correctly selecting the target picture from a four-picture array. These items were intended to teach the child how to use the response pad to make correct responses. The experiment began after the child responded correctly to two training items. If the child responded incorrectly to either of the first two training items, an easier training level was administered; however, all children in the study responded correctly to the first set of training items. After training, children heard a word presented at 70 dBHL through insert earphones (ER3A, Etymotic Research Inc., Elk Grove Village, IL, USA) and were asked to select one picture from the array that best illustrated the meaning of the target word by pressing the corresponding button on the response pad. Pre-recorded words voiced by an English-speaking female recorded at a rate of 250 Hz were presented prior to the four-picture array. Each picture of the array has a rectangular frame (monitor size was 17′′ and each colored frame was 5′′ × 6.5′′) corresponding to the button color at the same spatial location on the response pad as shown on the monitor (see **Figure 1**). Each color-coded button on the pad had an equal probability of response (25% of 24 words per block). Each new trial was self-initiated by pressing any button on the response pad. The task was programmed to discontinue when three consecutive errors occurred and the final set was considered the maximum performance level assigned to the participant.

The stimulus sets were arranged in order of decreasing concreteness and increasing abstractness (i.e., norm-based critical range going from concrete to more abstract and complex) so that the task could be calibrated to the child’s vocabulary level as assessed by the norm-based standardized critical range. For instance, in Level 1, the child would be provided with pictures of the words Ball, Dog, Spoon, and Foot whereas at Level 5, the child would be given more abstract words such as Peeking, Sawing, Envelope, and Ruler. In this context, difficulty is not a confounding variable but rather it represents how abstractness has been traditionally operationalized, specifically in the field of experimental cognitive psychology, for behavioral measurement (for review see Paivio, 2007). Importantly, the administration was arranged such that order of presentation and concreteness (abstractness) level were not systematically related. Supplementary Material Table S1 presents all the stimuli used in the WVT task including targets and distracters.

Following the original design of the PPVT III administration and referring to the PPVT norms (see Footnote 1), our protocol used the initial practice trials to place children at the starting age-appropriate level. Therefore, completion of Levels 1–3 was variably distributed across our sample, with most children skipping Levels 1 and 2. However, our participants were given Levels 4, 5 and 6, from relatively easy/concrete to relatively more difficult/abstract. In a preliminary external validity analysis, each word was mapped within all and each level included in the PPVT database to an independent corresponding set of concreteness values taken from the MRC Psycholinguistic Database (Wilson, 1988). Analysis of variance (ANOVA) analyses revealed that overall there was a clear very strong inverse linear trend indicating that concreteness drops systematically as difficulty level progresses [from approximately a frequency of 600 per 1000 words at Level 1 to 560 by Level 6; *F*(1,138) = 9.02; MSE = 2202.02; *p* < 0.01]. However, a more focused contrast analysis also revealed that while the mean concreteness level of the body of words collected under Level 4 (*M* = 577.48; SD = 37.04) was significantly higher than the body of words collected under Level 5 (*M* = 551.06; SD = 67.29) and Level 6 (*M* = 560.35; SD = 55.71), the latter levels did not significantly differ from each other [*F*(1,34) = 0.20, *p* = 0.66]. Therefore, Levels 5 and 6 were collapsed together in all following analysis and systematically compared against Level 4. The latter data reduction was further confirmed by other analysis showing that mean concreteness for Level 4 was significantly higher than for the combined Levels 5 and 6 [*F*(1,63) = 2.10; MSE = 2202.02; *p* < 0.05]. For the sake of both presentation and interpretation, in what follows we will refer to Level 4 as “concrete” words condition, and Levels 5 and 6 as “abstract” words condition.

#### EEG Data Acquisition and Recording

Children were tested individually in a sound-proof electromagnetically shielded EEG booth and reminded of instructions prior to each counter-balanced task. The child could communicate with experimenters and attending parents, in the adjacent control room, through an intercom speaker system. Parents and experimenters had full view of the child throughout the tasks by means of a bluetooth camera and booth window. Children were reminded throughout the tasks of the importance of not speaking, moving, turning the head, clenching teeth, or blinking during or after the manual response or before initiating a new trial in order to reduce movement and blink artifacts. Practice trials were completed just prior to their respective experiment task while children’s EEGs were recorded.

Electrophysiological signals were amplified (gain of 10; range of ±200 μV, or 400 μV peak-to-peak; Accuracy 29.80 nV/LSB) and low pass filtered at 500 Hz via SynAmps RT with a sampling rate of 1000 Hz. Acquisition filters were single-pole Butterworth, 6 dB per octave, 3 dB down at 500 Hz. All electrodes were referenced to a separate reference electrode and all data were re-referenced to a common average reference (i.e., the average was subtracted from each electrode for each time point). For post-data acquisition, ERPs were averaged separately for each stimulus type and condition for each electrode with an epoch of -200 ms prestimulus to 1000 ms post-stimulus. Trials contaminated by excessive peak-to-peak deflection (i.e., >100 or < -100 μV) at non-ocular electrode sites were excluded from the average. The proportion of rejected trials was less than 10% after artifact correction and removal. The post-data acquisition average signal from the electrodes was amplified and digitized with filter settings at 0.15 Hz (high pass) and 100 Hz (low pass).

The EEG recordings were obtained from electrodes held in place by means of an elastic cap (Quik Cap, Neuroscan, El Paso, TX, USA) adhering to the 10–20 system of measurements (American Electroencephalographic Society Electrode Position Nomenclature Committee, 1991) with 32 recessed Ag–AgCl electrodes (10 mm each) mounted in an electrode cap amplified (0.5–250 Hz sampled up to 1000 Hz) and referenced to a separate reference electrode located on the nose tip with AFz ground. The EEG montage was based on external landmarks of the skull (i.e., the inion, the nasion, the left and right preauricular points) and the electrodes were distributed based on percentage distances from these reference points. The channel configuration included a subset of 14 electrodes from a configuration at Frontal (F3, Fz, F4), Central (Cz), Temporal (T7, T8), Parietal (P7, Pz, P8), and Occipital (O1, Oz, O2) sites, plus one bipolar eye channel (HEOG) and a Nose reference. As detailed in the introduction, these electrode sites mapped onto the four key developmental brain organization subdivisions used as the underlying framework (Coch et al., 2005; Stevens et al., 2008). The decision to have a relatively small number of electrodes (<32) was dictated by convenience, duration, and possible fatigue and boredom effects raised by the ethical boards that approved the study. Nevertheless, previous preliminary work and pilot studies with the same number and configuration of electrodes showed no critical loss of reliability in ERP analysis results (Van Roon and D’Angiulli, 2014).

Horizontal eye movements were monitored with electrodes from a split bipolar electrode positioned at the outer canthi at HEOG. All impedances were kept under 5 kΩ so that they provided effective electrical signal with minimal noise. ERPs were recorded from the participants during the entire duration of all tasks. Resting EEGs (i.e., pre and post-experiment 2-min open/closed eyes) were clinically unremarkable in all children.

The electrode locations were mapped and analyzed using Brain Electric Source Analysis (BESA v.5.4.28), an electroencephalographic analysis software package. Ocular correction was assessed using PCA and the BESA Surrogate Model (BR_Brain Regions_LR.bsa). Data from two children were partially incomplete (one child completed Level 5/6 of the WVT and the other child completed Levels 4 and 5, but not 6) and presented very noisy background EEGs. In order to include these data, additional manipulations were needed. Specifically, ~15% of their data were interpolated using modeling based on grand averages so that they could be included in the graphic displays of the ERP waves (shown in **Figure 2**). For these additional manipulations, in addition to BESA, we also used routines available in the EEGLAB software (Delorme and Makeig, 2004).

Separate epochs for the auditory and visual part of the WVT task were averaged. The auditory task was averaged at the onset of aural word presentation if the response to the visual stimulus was correct. The visual stimuli were also averaged from the onset of the four-picture array if the response to the visual stimulus was correct. Baseline correction was based on a 200 ms pre-stimulus interval. The mean number (and standard deviation) of valid epochs for the auditory part of the WVT for Level 4 and Levels 5 and 6 were, respectively, 21.64 (SD = 2.34; *n* = 12) and 46.73 (SD = 3.18). The mean number (and standard deviation) of valid epochs for the visual part of the WVT for Level 4 and Levels 5 and 6 were, respectively, 18.82 (SD = 2.03; *n* = 12) and 37.91 (SD = 2.69). The relative robustness of the obtained “grand averages of subjects averages” was double-checked by conducting “omnibus” grand averaging of the total valid and corrected trials/epochs irrespective of subject (that is, lumping together related observations on the assumption they are practically independent) so that, for WVT-Visual, grand averages were taken on grand totals of 226 and 493 epochs for Level 4 and Levels 5 and 6, respectively, and for WVT-Auditory grand averages were taken on grand totals of 260 and 607 epochs for Level 4 and Levels 5 and 6, respectively. There were no substantial differences between grand averages of subjects averages vs. omnibus grand averages, the wave patterns were very similar (bin by bin correlations were *r* > 0.87). Thus, in spite of the relatively small number of trials by subject, particularly for Level 4, individual differences or variability really did not have a major impact on the pattern of the data.

#### ERP Data Processing

Time series analysis of the ERP data points was simplified by using a standard binning procedure (as in Berman and Friedman, 1995). Bins of 100 ms were determined for the entire epoch (-200 to 1000 ms) and the amplitudes were assessed for all electrode sites. Average waveforms for all 14 channels were output to Excel (Microsoft Office Professional Plus, version 14.0.7128.50000) and defined into averaged 100 ms bins resulting in 12 bins of 100 ms each, where peak activity occurred within the bin and with the first two bins representing the prestimulus baseline. However, to exclude confounding effects due to motor response in the WVT-V the analyses focused only on bins that could be safely assumed not to be affected by motor response. Therefore, the overall range of RTs across the sample was established as the temporal range in which the motor response overlapped with the processes of interest. In the WVT-V, the RT range was 605.73–3566.73 ms for the concrete words condition (PPVT4), and 2104.92–4651.02 ms for the abstract words condition (PPVT5 and 6)^2^. The resulting bin range was 0–999 ms because the prestimulus interval of -200 to 0 ms was used to determine the region where no task activity occurred (Woodman, 2010). No response was required for the WVT-A, therefore the bin range was kept consistent with WVT-V. Critically, our analyses focused on early (0–299 ms), intermediate (300–699 ms), and late (700–1000 ms) ERP intervals which did not overlap with any RT range in the WVT-V. The ensuing analysis, which was based on the binned ERP data, entailed a *by*-*item* analysis approach. That is, for each electrode, the twelve bins, collapsing means across all children, were treated as random variables, and the generalizability of results therefore referred to both subjects and items populations (see Bedny et al., 2007).

## Results

### Behavioral Data

Accuracy in the WVT was relatively high as all children completed successfully between 2 and 6 sets of target words, all children scored within ±1 SD from the mean in terms of age-normed standard scores. Recall that specific accuracy measures were not assessed as the task was structured such that three errors in a row would end the task. All participants except one were able to complete up to Level 5 or 6 of the PPVT. Response times (RTs) were significantly faster (*t*_(10)_ = -2.447, *p* < 0.05) for the PPVT 4 (*M_RT_* = 2587.47 ms, SD*_RT_* = 791.14) than for the combined Levels 5 and 6 (*M_RT_* = 3243.38, SD*_RT_* = 786.92). Recall that Levels 5 and 6 were combined as there were no significant differences in RT between these conditions. There was no correlation between age and RTs. Behavioral measures of performance and reaction times and patterns of ERP activity showed that children carried out the auditory and visual parts of the WVT in a similar way.

### Control Analyses for Order Effects and Associative Relationships

Curve-fit estimations which simultaneously tested the following regression functions: linear, quadratic, exponential, growth curve, logarithmic, inverse, and power – were performed on the presentation of the stimuli to exclude presentation order effects in the data acting as latent confounding variables. The analyses were run on the entire set of stimuli and subsequently split by level. None of the 56 multiple analyses proved significant thus indicating no effect of presentation order for any of the conditions either alone or grouped (i.e., on a binomial test, the chance of having all these regression coefficients simultaneously below significance is *p* < 0.0001).

A second control analysis consisted of multiple regressions to test possible associative relationships of ERP with control variables such as age, accuracy, and RTs on the tasks, subjects’ profiles used for screening, and sample selection (C-TRF, CBCL, BRIEF-P). All effects were not significant (all *p* values > 0.05). These results showed that the main findings likely were not due to confound of these other factors and that the responses of the children were typical for the preschool age range.

### ERP Data

#### Mean Amplitude Differences by Bins

Bin parsing of continuous ERP data (see ‘ERP data processing’ section) permitted to compare *absolute* amplitude differences between concrete and abstract conditions in *spatial* location and *temporal* interval. This approach also permitted to examine the extent of selectivity of the early and late ERP activity, and to examine effects specific to the tasks common to both concrete and abstract conditions, against background random effects unrelated to the tasks. In other words, this particular analysis had the goal to show the significant “abstract-concrete wave differentials” (something akin to a “microvolt measuring stick”) across the ERP epochs, as well as the corresponding polarity direction effects (see **Figure 2A**).

Thus, across the entire ERP epoch, the first analysis involved determining the minimum significant microvolt difference for either: (1) the absolute differences between the average waveform data points in case of iso-polarity effects (see subpanel 1 in **Figure 2A**), or (2) the absolute distances between the average waveform data points in case of reverse-polarity effects (see subpanel 2 in **Figure 2A**). Specifically, focused ANOVA contrasts between the paired binned mean amplitudes were conducted so that the minimum significant standardized absolute difference was derived using the following formula (as in Rosenthal et al., 1999):

$$Abs\,\;amp\,\;diff_{\mu V}=\left|M_{ERP}\lambda_{i}\right|=t_{crit}\cdot \left(\sqrt{{MSE}_{within\cdot }\left(\overset{\lambda_{i}^{2}}{\underset{\overset{¯}{n_{i}}}{\sum }}\right)}\right)$$

Where, for each bin interval *i*, M_ERPi_ indicates the predicted mean difference between pairs of same time-interval bin microvolt values, λi represented the contrast weights, *t*_crit_ represented the *t*-value corresponding to the *p*-value in the two-tailed Student’s *t* Probability Density Function, determining the critical significance threshold at 0.025 [*t*_crit_(12) = 2.56, two-tailed], after the critical *p*-value was corrected for multiple comparisons using the Simes–Bonferroni procedure (Simes, 1986). The MSE_within_ represents the error factor entered in the focused *t*-contrasts across all comparisons (this was the largest and most conservative Within-Subjects Mean Square Error at the highest-level, three-way, interaction, Domain general × Domain specific × Condition, derived from the linear contrasts (WVT-V: MSE = 0.85; WVT-A: MSE = 0.77) that takes into account the largest possible variance of the baseline mean across the entire ERP epoch following the general recommendations by Rosenthal et al. (1999). Accordingly, the initial analysis included separate mixed-model (Type III SSE) four-way ANOVAs for the Auditory and the Visual parts of the WVT, with the following factors: Domain general (within-subjects: F, C/T, P, O) × Domain specific (within-subjects: Right, Midline, Left) × Condition (within-subjects: Concrete vs. Abstract) × Bin (between-subjects: 12 100-ms bins). For the present and all the following analyses, within-subjects effects were adjusted using the Greenhouse–Geisser correction. For both WVT tasks, the three-way interaction *Domain general × Domain specific × Condition* was significant [Auditory: *F*(1,144) = 4.14; MSE = 4.00, *p* < 0.01; Visual: *F*(1,144) = 19.32; MSE = 4.89, *p* < 0.001].

To determine the background ERP activity of non-interest, multiple focused contrast comparisons were computed for the absolute amplitude difference in the first two bins in both tasks, which represented the 200 ms prestimulus interval and were used to assess a period during which the participant’s brain activity was not performing the task. Differences at prestimulus were not significant across conditions [*t*_contrast_(12) < 1].

#### Peak Amplitude Analysis

Next, we conducted a bin-by-bin automatic (“blind”) peak amplitude analysis procedure, whereby, the effects were quantified via standardized differences (i.e., focused *t*-contrasts corrected for multiple comparisons) across the epochs. **Figure 2A** explains graphically the type of effects studied in our analysis of the observed waveform patterns. As a result of all these steps, to be judged as a valid effect, the value of each standardized mean wave difference of the data bins had: (a) to be statistically significant, (b) to agree with a corresponding significant peak-to-peak difference (identifiable in **Figure 2**), and (c) to exceed the not-of-interest background ERP activity differences. Therefore, the determination of the “true” (valid) effects was established through the fulfillment of converging non-arbitrary criteria.

**Figures 2B,C** shows averaged ERP waveforms for concrete and abstract words conditions in the auditory and visual parts of the WVT, respectively. Inspection of the waves for the Auditory part of the WVT (**Figure 2B**) shows early ERPs (<200 ms) in the temporal areas, especially the left side, followed by a P300-like waveform in mid parietal and Frontal, especially left side, with subsequent late activity in the temporal and occipital, especially in the midline, starting approximately from 600 ms. The described pattern seems relatively clear during the concrete words condition, but it is much less pronounced for abstract words. The waves for the Visual part of the WVT show early ERPs in Left Temporal-Parietal as well as the Left and midline Occipital sites, followed by late ERPs again the Left Temporal-Parietal sites and all the electrodes across both Midline and Right side.

**Table 2** reports the significant mean amplitude differences (and standard errors) from pairwise focused contrasts in the interaction between Domain general, Domain specific and the Early, Intermediate, and Late ranges of binned ERP waveforms in the visual (Top Panel) and auditory (Bottom Panel) parts of the WVT. The reported mean amplitude effects correspond to the peaks shown in **Figures 2B,C**.

**Table 2:** Mean amplitude differences of Early (0–299 ms), Intermediate (300–699 ms), and Late (700–1000 ms) intervals of binned ERPs in the visual and visual parts of the word verification task (WVT).

	ERP waveform		
	Early	Intermediate	Late
**Visual WVT**
**Frontal network**
Left (F3)	2.91 (0.36) ^C+^	2.20 (0.56) ^C+^	2.48 (0.45) ^A+vs.C-^
Midline (Fz)		2.91 (0.30)	3.33 (0.38) ^A+vs.C-^
Right (F4)	2.30 (0.50) ^C-^	2.36 (0.45) ^C-^	2.60 (0.37) ^A+vs.C-^
**Centro-temporal network**
Left (T7)	2.57 (0.34) ^C-^	2.28 (0.17)	2.47 (0.32) ^A-vs.C+^
Midline (Cz)	4.35 (0.49) ^A+vs.C-^	2.97 (0.45)	
Right (T8)			
**Parietal network**
Left (P7)	3.18 (0.42) ^C-^	2.37 (0.30)	3.22 (0.46) ^vs.C+^
Midline (Pz)	3.88 (0.44) ^C-^	2.39 (0.33) ^A+^	
Right (P8)	3.19 (0.39) ^C+^	3.15 (0.58) ^C+^	3.29 (0.64) ^A+vs.C-^
**Occipital network**
Left (O1)		2.45 (0.33) ^C-^	2.55 (0.31)
Midline (Oz)	2.74 (0.39) ^C+^		2.81 (0.44) ^C-^
Right (O2)	2.80 (0.37) ^C+^		2.50 (0.41) ^C-^
**Auditory WVT**
**Frontal network**
Left (F3)			
Midline (Fz)		2.29 (0.49) ^vs.C+^	
Right (F4)		2.75 (0.57) ^C+^	2.61 (0.61) ^C+^
**Centro-temporal network**
Left (T7)		2.57 (0.91) ^C+^
Midline (Cz)			2.15 (0.43) ^C-^
Right (T8)			1.99 (0.58) ^vs.C+^
**Parietal network**
Left (P7)		2.69 (1.27) ^A+^
Midline (Pz)	3.34 (0.68) ^A+^	1.90^†^ (0.69) ^A+ = C+^	3.97 (1.07) ^C+^
Right (P8)		2.42 (0.93) ^C-^	2.21 (1.34) ^A+^
**Occipital network**
Left (O1)			
Midline (Oz)		2.65 (1.00) ^C-^
Right (O2)		3.01 (1.06) ^A+vs.C-^

*All values within the table are in microvolts. Iso-polarity differences in favor of concrete words are denoted with the ^C^ superscript, while difference effects in favor of abstract words are denoted with the ^A^ superscript. The signs + and – in superscript denote the polarity of the ERP deflections. Reverse-polarity differences are denoted with ^A ± vs. C ±^ superscript. Overlapping iso-polar waveforms with same phase are denoted with ^A ± = C ±^ superscript (see also **Figure 2A**). †*p* = 0.05.*

#### Contrast Analysis by ERP Intervals

In a more compact model, reducing the complexity of the ANOVA design, we used as dependent variable the absolute value waveform differences (i.e., difference scores). The primary analysis included a mixed-model (Type III SSE) four-way ANOVA with the following factors: *Task* (within-subjects: Auditory vs. Visual) × *Domain general* (within-subjects: F, C/T, P, O) × *Domain specific* (within-subjects: Right, Midline, Left) × *ERP Interval* [between-subjects: Early (0–299 ms), Intermediate (300–699 ms), Late (700–1000 ms)]. Tests of linear polynomial trends yielded significance for the within-subjects effects of Domain general [*F*(1,127) = 4.23; MSE = 10.36. *p* < 0.05] and of Domain specific [*F*(1,127) = 10.66; MSE = 10.36; *p* < 0.01], as well as main effect of Task [*F*(1,127 = 22.16; MSE = 5.69; *p* < 0.0001]; in particular, the latter result showed that increased ERP activity for concrete as compared to abstract words was significantly higher in the visual rather than in the auditory WVT. However, in the early and/or late interval, ERP activity was consistently higher than in the intermediate interval [between-subject effect of ERP interval: *F*(2,127) = 3.68; MSE = 2.31; *p* < 0.05] and this pattern did not vary in relation to the type of task (*F* < 1). The interaction between Task, Domain general, and Domain specific was significant [*F*(2,127) = 6.82; MSE = 3.93; *p* < 0.05], as it was the interaction between Task, Domain general, and ERP interval [*F*(2,127) = 4.45; MSE = 14.76; *p* < 0.05] and the interaction between Task and Domain specific [*F*(1,127) = 17.94; MSE = 4.10; *p* < 0.0001]. The interaction between Domain general and Domain specific was significant when the contrast term corresponding to Domain specific fitted a quadratic trend [*F*(1,127 = 3.76; MSE = 3.93; *p* < 0.01]. No other effect was significant.

#### ERP Activity Pathways Analysis

**FIGURE 3:**
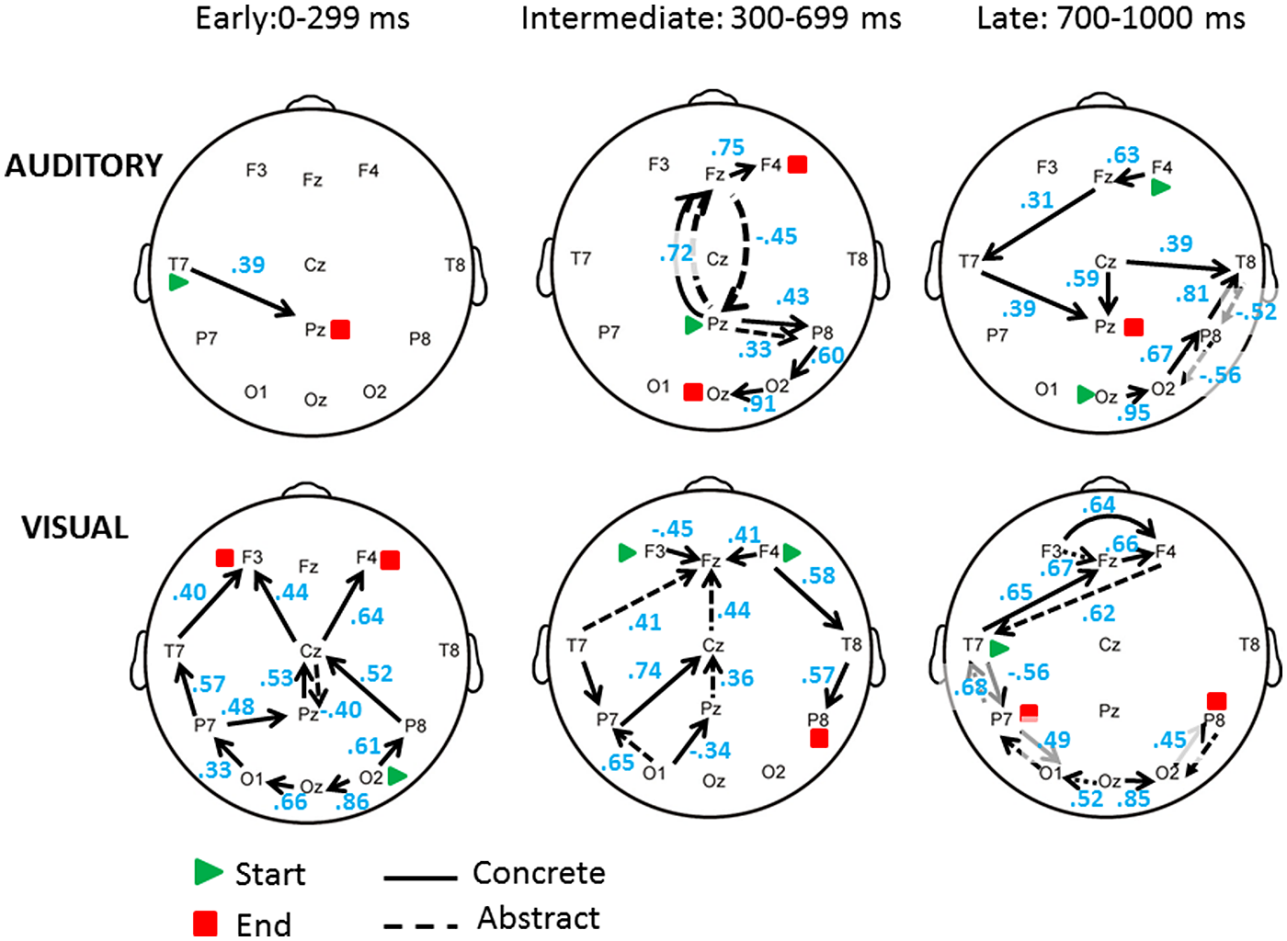
**Event-related potential activity pathways analysis summarizing the spread (least-path connections) of observed scalp grand average ERP activity from one electrode/area to the next.** All correlations reported (shown in blue) were significant (*p* < 0.05) after multiple comparison correction.

Although the outcomes of the ANOVA analysis mainly agree and confirm the patterns of the means shown in the other types of analyses, given their complexity, the findings can be more easily understood by means of graphic summary. **Figure 3** offers a synthesis of all present converging data detailing the spread of observed scalp grand average ERP activity from one electrode/area to the next. To obtain these summary activity maps, four basic rules were followed. First, the information relative to the significant ERP differences observed in **Figure 2** and the corresponding means in **Table 2** were combined and were plotted on head maps sequentially – i.e., according to the actual ordered timing of independent occurrence of each effect/peak as measured ERP latency– broken down by the three ERP intervals examined; if more than one peak per electrode/area occurred at the same time (non-independent), they were all plotted as spreading at the same time to the next significantly active electrode/area. Second, the principle of *neural wiring minimization* (Cherniak, 2012) was used as a rationale for joining the least-path connections between significant ERP activity electrodes/areas. Third, the direction of the spread of activity – i.e., forward, backward, or lateral –from one electrode/area to another one was determined by integrating all available contextual clues. That is, by integrating: (a) the information gathered by applying the previous two rules, plus (b) the direction or sign of the pairwise Pearson correlations between electrodes’ activity [direct (positive) correlation was interpreted as excitatory activity, inverse (negative) correlation was interpreted as inhibitory activity; note: the correlation coefficients are shown in blue in **Figure 3** for each pairwise electrode connection; see also notes in figure caption]; and lastly (c) the two additional constraints that for the occipital electrodes (O1, Oz, O2) the permissible spread to next electrodes could be only forward or lateral, and similarly for frontal electrodes (F3, Fz, F4) the permissible spread to next electrodes could be only backward or lateral, for the obvious neuroanatomical design. Fourth, within each ERP interval, the earliest and latest significant differential effects as shown in **Figures 2B,C** were designated as the start and end point of the activity propagation relative to the ERP epoch.

As shown in **Figure 3**, during the early part of the auditory WVT there was a significant response to concrete words in the proximity of the auditory temporal left region followed by activity in the midline parietal region. In the intermediate period, the ERP activity of interest seemed to occur in response to both concrete and abstract words across the midline involving the cross-talk between mid-central, frontal and lastly ending up at the occipital sites. During the late period of the auditory WVT, ERP activity seemed to involve mainly the posterior right side, but also some of the same midline and left sites that were activated in the earlier intervals.

During the early part of the visual WVT, ERP activity mainly related to concrete words involved occipital and parietal sites seemingly propagating from the posterior to the anterior left side regions. Then, by the intermediate period of the task, processing for both concrete and abstract words seemed to progress from frontal left-side areas to left posterior, especially occipital, as well as right temporal areas for concrete words. Lastly, late processing seemed to involve the left side sites similarly for concrete and abstract words, with significant activity in the occipital and frontal areas but ending in the left parietal region.

#### Analysis of Correlations between Auditory and Visual ERPs

Following the pathway analysis, we ran multiple simultaneous pairwise correlations to examine whether the early and late auditory ERP activity in the areas involved in the domain general functions would correlate with the early and late visual activity in the same functional sites. Thus, the sets of correlations were computed among the mean ERP data for each subject grouped by ERP interval, and included four groups of electrodes (O, P, C-T, F) for concrete and abstract words conditions. The rate of total significant correlations was 17 against the total rate of 1024 of significant correlations comparing all electrodes in the set of all possible comparisons, including the irrelevant ones. As assessed by binomial test, for our subset of tests, the likelihood of obtaining a significant correlation (with *p* < 0.05) by chance due to multiple testing corresponded to a *p* < 0.00001. In what follows, we report only the significant findings.

The correlations between the auditory and visual ERP activities were proportionally and significantly less in the concrete than in the abstract condition (0.31 vs. 0.81, *Z* = 2.85, *p* < 0.01). Direct correlations indicate that more of similar processing linked with the auditory part, also occurred during the visual part of the task, whereas indirect correlations mean that less processing concurrent with the visual part occurred when there was more auditory activity for the same functional areas. Early auditory ERP activity was correlated to early visual activity only for the abstract condition. An inverse (negative) correlation was found for Frontal [*r*(13) = -0.59], Occipital [*r*(13) = -0.73], and Parietal [*r*(13) = -0.72] regions; however, we also found a direct (positive) correlation for Central-Temporal regions [*r*(13) = 0.57]. Early auditory activity was also inversely correlated with late visual activity only in Parietal regions [*r*(13) = -0.65] for the concrete condition, but again a different pattern was found for the abstract condition whereby Occipital, Parietal, and Frontal sites showed direct correlations [*r*(13)’s: 0.57, 0.61, 0.67, respectively] but Central-Temporal regions showed an inverse correlation [*r*(13) = -0.71]. A pattern similar to the latter finding but involving just Parietal and Central-Temporal areas was found between late auditory and early visual activity: for the concrete condition, P electrodes showed a direct correlation [*r*(13) = 0.78] but the C-T electrodes showed an inverse one [*r*(13) = -0.66]; the opposite, albeit with larger effect sizes, was observed for the abstract condition [P: *r*(13) = -0.84; C-T: *r*(13) = 0.77]. Finally, auditory and visual late activities were related inversely in Parietal [*r*(13) = -0.81] and directly in Central-Temporal [*r*(13) = 0.73] regions for concrete condition, whereas for the abstract condition, Occipital, Parietal, and Frontal regions all showed direct correlations [*r*(13)’s: 0.65, 0.76, 0.63, respectively].

Although, there was no consistent correspondence between a specific pattern of correlations and the conditions, overall these results suggest that the ERP activity during the auditory part of the WVT was generally related to the subsequent activity during the visual part.

## Discussion

Event-related potential correlates underlying word-verification, with separate ERP averages for correct responses to auditory and visual stimuli, were used to examine the dynamics and pathways for an embodied cognitive approach to visualization in preschool children. *Temporally* speaking, the findings indicate that during auditory and visual word verification early ERP activity (i.e., 0–299 ms) was mostly devoted to the processing of concrete words and the processing of abstract words became evident only at intermediate and late ERPs. *Spatially* speaking, while early ERPs in the auditory task were linked to left temporo-parietal activity for concrete words, the visual task elicited occipito-parietal activity for concrete words and centro-parietal activity for abstract words. In intermediate ERPs (i.e., 300–699 ms), the auditory task led to parieto-occipital and parieto-frontal activity for the processing of both concrete and abstract words, while in the visual task a parieto-central activity was common to both types of words. In the case of late ERPs (i.e., 700–1000 ms), the auditory task involved a right-hemispheric ant-posterior pathway activation of occipital, parietal and temporal areas during the processing of both abstract and concrete words, while the visual task required left-hemispheric post-anterior activation (i.e., frontal, temporal, parietal, and occipital areas) for the processing of both types of words. The differences in the ERP activity pattern suggest that children generally recruited neural dynamics that are typically recruited during visual perceptual processing (i.e., matching visualization). Although the embodiment approach seems to contribute to the advancement of our understanding of children’s concrete and abstract word processing in terms of interplay among motor, memory, and sensory-perceptual networks, there have been no previous attempts to fully specify the corresponding neural dynamics.

In terms of neural activity (see **Figures 2B,C**) and dynamics (see **Figure 3**), the present findings indicate that processing patterns in response to the concrete and abstract words were similar up to a point in the first part of the task. Although both concrete and abstract words, presented aurally, were processed parieto-frontally and parieto-occipitally during the intermediate interval (300–699 ms), concrete words also activated right frontal areas (F4). ERP research shows that decision-making (generally coinciding with parietal and then frontal activation) for relevant stimuli typically occurs within a temporal interval distribution having as a median 300 ms, but possibly occurring later in very young children and difficult to distinguish (for review see Andreassi, 2007). For the concrete words, the children appear to have made a decision and subsequently ‘imaged’ the word. Similar parieto-occipital activity occurred in response to abstract words; however, abstract words also activated the right temporal site prior to decision-making and followed by frontal sites suggesting, perhaps, metaphorical encoding (Lakoff, 2014) of the abstract words, and decision-making, followed by executive functions. In the later period of the auditory part (700–1000 ms), concrete words continued to show right occipital activity followed by parietal and temporal activity, whereas abstract words continued to show a reverse pathway of activity (T8→P8→O2). The sequence of late activity for the concrete words suggests that the child might have continued visualization prior to presentation of the visual picture array, accessing linguistic representations from left frontal, and associative and sensori-motor memory from the right temporal and the posterior regions in the right-hemisphere.

The visual picture array of the concrete and abstract words directly followed the auditory presentation of each word. During the early interval (0–299 ms) of this visual processing, concrete words continued to show occipital activity (O2, Oz, and O1), whereas abstract words activated the midline parietal (Pz) electrode, suggesting more visualization for the concrete words and the beginning of an embodied component for the abstract words early on. The continuation of visual processing in this second part of the task is reasonable when we consider the prior auditory presentation of the word. A similar type of elaboration seemed to continue during the intermediate interval (300–699 ms), when the concrete words activated the top-down fronto-temporo-parietal response of the right-hemispheric areas (F4, T8, P8) and the temporo-parieto-occipital network in the left-hemisphere, while abstract words activated the response of midline Pz-Cz-Fz areas and some left occipito-parietal areas. According to our proposed neurocognitive framework, the ante-posterior activity in the right-hemisphere and the posterior activity in the left-hemisphere associated with the concrete words suggest the involvement of executive, memory and visuo-spatial working memory networks which support the generation of visual mental imagery (Kosslyn, 1994; Kosslyn et al., 2006). In contrasts, the temporo-parieto-occipital activity in the left-hemisphere associated with the abstract words would resonate with the engagement of linguistic but also visuo-spatial working memory and the attentional networks. This pattern would lend support to some of Borghi’s and other authors’ contentions concerning the predominant involvement of linguistic and metaphoric elaboration in comprehending the meaning of abstract words (see our discussion below), including the related idea that both these processes (for imagery and language) may exploit or “reuse” neural mechanisms evolved and developed for regulating social interaction via imitation, planning and execution of actions (Gallese, 2008; D’Angiulli and Schibli, 2011).

For the duration of the late interval, concrete words followed two, presumably related pathways: an interactive top-down sequence of activity from left Temporal (T7) to right Frontal (F4) and, another sequence of activity again from left Temporal to left Parietal (P7) then connecting to all occipital sites, response which could be an indication of the child’s sustained imagery of the word. The abstract words during the 300–699 ms period activated left parietal (P7) and left occipital (O1) electrodes. We speculate that the activity taking place in the later interval (700–1000 ms) might have, therefore, involved what Craik and Lockhart (1972) refer to as a *deep level of processing*. It could be entertained that this is perhaps indicative of further embodiment processing in that as deep processing began for the abstract words, occipital activity was predominantly observed, implying that the child might ‘visualize’ the word during comprehension. In short, the findings would suggest that for concrete words, it could be that the child generated a visual mental image corresponding to the object described by the word relatively quickly, subsequently just continued ‘thinking’ about it. In contrast, for abstract words, the child first ‘embodied’ the word, then ‘visualized’ it.

Our data showed that the vision-language integration for single-word comprehension works similarly whether words are concrete or abstract. However, our findings also suggest that, paradoxically, more visual information is needed in using and comprehending abstract rather than concrete words as shown by the continuous occipital activation occurring during deep processing (comprehension) possibly due to the way in which the latter words are acquired. Previous research (see for example Borghi et al., 2011) suggests that abstract words involve more in-depth complex visualizations and, therefore, require more cognitive control and effort during elaboration (i.e., the late part of the ERP from 700 to 1000 ms). Specifically, elaboration for abstract words requires more complex relations (e.g., social interactions) unlike single item processing for concrete words where significant co-activations showed much more frontal activity during abstract compared with concrete word processing.

In terms of patterns of correlated early and late activity for homologous areas in the auditory and visual parts of the task, the pattern of correlations were very similar, albeit more pronounced for abstract words. Although, there was no consistent correspondence between a specific pattern of correlations and the conditions, overall the data indicated that the ERP activity during the auditory part of the WVT was generally related to the subsequent activity during the visual part. These results coalesced with the dynamic interplay hypothesized by DIVLA where simulations based on auditory and visual-linguistic processes “guided” each other and were progressively updated in a similar fashion whether processing was auditory or visual.

As predicted, our results support the DIVLA hypothesis of a perceptual-motor model rather than the motor model proposed by strong embodiment theories. In both auditory and visual parts of the task, the observed ERPs showed involvement of frontal premotor processes at 700–750 ms, which cannot be explained as preparation for response to the picture selection required by the visual (matching visualization) part. Furthermore, the observed central activation patterns do not support a motor model because the task may reflect an aspect of word comprehension in children that, to our knowledge, has not been adequately investigated by other studies in the embodiment literature. The emphasized aspects do not necessarily require a manual or overt response (for a similar point see Mahon and Caramazza, 2008). Instead our study indicates a more widespread and integrated activation which is more representative of a low embodiment (or even disembodied) view. Hence, new aspects of embodiment need to be integrated into the corpus of evidence and data already accumulated in the research of early childhood linguistics.

Another striking aspect highlighted by the data is that, in both auditory and visual parts of the task, visualizations seem similar from the point of view of late neural responses implicating posterior brain structures which typically process visual objects even for aural word presentation. Our results support a vision-language interaction based on *a supramodal map of perceptual salience* (Spivey, 2007). That is, a form of mapping that puts in correspondence auditory and visual information, presumably in the parietal cortex, and then forms a unitary merge of the two sources of information preserving the distribution of salience of the individual inputs. A salience map is able to feed back to and influence the functioning of uni-modal sources. While visuo-auditory matching visualizations (WVT-V) support multisensory integration between auditory-linguistic and visual-object modalities, post-auditory (WVT-A) visualizations support an integrated map of multiple memory traces in the *episodic buffer* (Baddeley, 2000; D’Angiulli et al., 2013). All the mechanisms involved provide detailed knowledge of the processes postulated by DIVLA and could be incorporated into the context of that theoretical higher-level framework.

Although the visual and auditory processes addressed do not focus on social aspects of embodiment, they provide accounts of the “cognitively necessary” basis for social transactions that unfold during human development (see Piaget, 1965/1995) and, specifically, for socially acquired developmental domains such as word acquisition. Consequently, the development of word comprehension may rely heavily on non-linguistic, visual processes, regardless of the word’s ‘concreteness’ or ‘abstractness.’ The present study supports a developmental derivation of the DIVLA argument (Mishra and Marmolejo-Ramos, 2010) that word meaning acquisition in typically developing children is associated with the visual contexts in which words are learned. For example, in a recent study examining sensorimotor experience in blind children, Iossifova and Marmolejo-Ramos (2013) found that blind children were more likely than sighted or visual-motor impaired children to rely on their body as a reference frame when processing abstract concepts. Therefore, overall our findings support the view that the social transmission of word meanings to typically developing children depends on the attended visual experiences in the contexts in which words are acquired and learned.

As it could be said for many published reports, the present experiment could be improved further. We had a relatively small sample, the complexity of the visual part of our task (i.e., WVT-V) could be greatly reduced (i.e., singular presentation of auditory word and picture pairs, instead of an array from which the participant needs to select the correct response) to elicit more easily analyzable and interpretable brain responses, and perhaps a more recently normed, wider word database could be used. However, the limitations do not outweigh the important leading insights that our findings afford in the relatively understudied area of young (i.e., 3–5 years-old) children’s visualizations and their neural substrates (see D’Angiulli, 2011).

In summary, the present research contributes to defining an initial, more detailed structure of the social processes underlying word acquisition and meanings in terms of basic, mediating embodied neurocognitive mechanisms. The implications of our findings for the interplay between social and cognitive embodiment suggest that words are social tools, not only because they extend our own bodily spatial influence (see Borghi et al., 2013; Granito et al., 2015), but also because, as originally proposed by Vygotsky (1962, 1978), *the extra-cortical organization of higher mental function* through symbols and tools regulates at the same time inter-subjective communication and the individual’s cognitive activities. Particularly relevant to word and meaning acquisition is one of the concepts linked with Vygotsky’s Zone of Proximal Development; the principle of *scaffolding* (Bodrova and Leong, 2012) which, in simplified terms, describes a possible process through which children may be more likely to advance their current knowledge level with the support of another person or more knowledgeable other (e.g., teachers, parents, peers, experts) via social interaction. For instance, advancing a child’s vocabulary is accomplished by driving other people’s attention and visualization in a direction leading to the sharing of intended experience and knowledge. We need words to evoke images of the world that can be exchanged among interlocutors for different types of social communication; hereof, language instruction and vocabulary acquisition are not unique. Images, pictures, gestures and non-verbal behaviors are all embodied instruments of social communication that are constantly used, for example, in learning activities carried out by children (e.g., think about the enormous role of picture books in Western culture literacy). Indeed, as recent research has shown, hand gestures not only improve learning in children (Goldin-Meadow, 2014) but that also assist in providing a link between concepts and body’s kinesics (Cook et al., 2008; Cevasco and Marmolejo-Ramos, 2013) such that language comprehension and production have bodily correlates.

## Conclusion

The central message from our findings is that social transmission of word meanings to young children does seem to depend on neurocognitive embodiment processes. The attended visual experience embedded within the interactive social contexts in which words are acquired, learned, and remembered plays a crucial role.

## Conflict of Interest Statement

The authors declare that the research was conducted in the absence of any commercial or financial relationships that could be construed as a potential conflict of interest.

## Footnotes

1 The Peabody Picture Vocabulary Test (PPVT-III) is a standardized measure used to measure a child’s receptive vocabulary, word comprehension and semantic elaboration (Dunn and Dunn, 2007). The strong relationship between the processes measured by the PPVT-III and language comprehension (Carroll, 1993; Hiebert and Kamil, 2005) show correlations between the PPVT-III and kindergarten language comprehension are fairly strong (*Mdn r* > 0.65, *p* < 0.05, see Dunn and Dunn, 2007), therefore, performance on the PPVT-III would likely reflect the child’s preschool level of linguistic processing. This has been confirmed by studies in aphasiology (Eisele and Aram, 1993; Yampolsky and Waters, 2002; DeDe et al., 2003), intelligence (Ingram et al., 1998; Bell et al., 2001), and clinical neuropsychology (Rourke et al., 2002) in children and adults.

2 Only one participant had a response value below 2000 ms, therefore, mean replacement was used so that the response value would be similar to the 5 and 6 response range of the other participants. The value with mean replacement was 2206 ms.
