# Supplementary material for: Neural correlates of visualizations of concrete and abstract words in preschool children: a developmental embodied approach
Source: Front Psychol. 2015 Jun 29;6:856. doi: 10.3389/fpsyg.2015.00856 (PMC4484221; doi:10.3389/fpsyg.2015.00856)
Supplement: Supplementary file 1 [file Table_1.PDF]

Supplementary Material

Supplementary Table 1. Word lists presented for the auditory presentation prior to the visual image array.

Word	Letter Length	Spoken Length (ms)	Visual Distractors
Level 4 (Concrete Words)			
Farm	4	851	airport, moon, village
Penguin	7	696	duck, eagle, ostrich
Gift	4	789	duffel, cornucopia, garbage
Feather	7	770	horns, tail, paw
Cobweb	6	953	gears, sprocket, nautilus
Elbow	6	573	hand, wrist, neck
Juggling	8	803	fencing, clapping, archery
Fountain	8	861	pagoda, building, cathedral
Net	3	593	hook, lure, rod
Shoulder	8	719	hand, foot, chin
Dressing	8	740	walking, pouring, splashing
Roof	4	496	stairs, fence, doors
Zipper	6	678	tie, belt, shirt
Nest	4	639	hive, terrarium, cage
Mountain	8	789	canyon, rock, rock fence
Horn	4	729	maraca, ukulele, castanet
Pear	4	491	banana, lemon, orange
Yawning	7	764	tying, knocking, reading
Caterpillar	11	870	snail, octopus, spider
Chin	4	584	forehead, brow, nape
Pouring	7	682	dusting, decorating, plugging
Decorated	9	819	bread, hotdog, sausage
Triangle	8	900	quadrilateral, hexagon, diamond
Desk	4	713	bench, safe, hammock
M	6.21	729.25	
SD	2.11	122.40	
Level 5			
Peeking	7	574	hanging, hammering, hoeing
Ruler	5	583	scissors, easel, paints
Tunnel	6	500	trestle, ski slope, interstate
Branch	6	760	wheat, rutabaga, pinecone
Envelope	8	679	magazine, newspaper, money
Diamond	7	815	hexagon, irregular hexagon, cigar-shaped
Calendar	8	638	picture, map, cheque

Buckle	6	667	clothes pin, safety pin, scoop
Sawing	6	685	drinking, making the bed, brushing teeth
Panda	5	551	raccoon, skunk, porcupine
Vest	4	667	jacket, sweater, shirt
Arrow	5	506	yo-yo, bow, shuttlecock
Knee	4	514	ankle, wrist, toes
Donkey	6	653	stag, camel, lamb
Measuring	9	837	cleaning, testing, massaging
Huge	4	642	large, medium, small
Coin	4	618	bust, money, statue
Porcupine	9	900	hyena, tapir, hippopotamus
Tearing	7	613	boxing, stealing, ringing
Rectangle	9	761	pentagon, quadrilateral, irregular hexagon
Full	4	534	3/4, 1/2, ¼
Astronaut	9	880	mom, guard, entomologist
Ship	4	586	motor boat, sail boat, raft
Hook	4	555	net, lure, rod
M	6.08	654.92	
SD	1.84	117.04	
Level 6 (Abstract Words)			
Picking	7	634	peeling, pouring, cracking
Target	6	650	skateboard, tetherball, sled
Dripping	8	806	flowing, spraying, sprinkling
Knight	6	556	circus horse, horse statue, equestrian horse
Delivering	10	912	picking, reading, hugging
Cactus	6	863	bush, garden arch, tree
Dentist	7	1004	nurse, barber, doctor
Floating	8	714	swimming, jumping, diving
Claw	4	522	horns, hooves, wing
Uniform	7	866	office wear, winter coat, casual wear
Gigantic	8	986	large, medium, small
Furry	5	649	colorful, scaly, feathery
map	3	568	magazine, weather vane, head stone
Lock	4	648	key, spring, trowel
Package	7	711	plaque mounted fish, garbage, cage
Fruit	5	770	cookies, vegetables, radishes
Brain	5	667	heart, lungs, kidneys
Goat	4	630	wildebeest, llama, bull
Jewelry	7	778	picture, ladle, handkerchief
Statue	6	987	circus horse, knight, equestrian horse
Chain	5	651	keys, bells, ropes

Leaking	7	657	spraying, sprinkling, flowing
Cashier	7	795	programmer, mail person, dishwasher
Binoculars	10	1066	headphones, microscope, stethoscope
M	6.33	753.75	
SD	1.79	153.80	
		Level 5&6 (Abstract Words)	
M	6.21	704.33	
SD	1.80	144.13	

Note. Each word was presented for a maximum of 2000 ms followed by a 10000 ms cross fix prior to the onset of the image array. The letter count for Level 4 – i.e., the concrete words condition (M=6.21, SD=2.11) versus Level 5&6– i.e., the abstract words condition (M=6.21, SD=1.80) was not significant $F(2, 71)=0.10$, $p=0.90$. The spoken word length for Level 4 (M=729.25, SD=122.40) versus Level 5&6 (M=704.33, SD=93.92) was also not significant $F(1,47)=0.63$, $p=0.43$.
